# Diagnosis and Management of Peritoneal Metastases from Ovarian Cancer

**DOI:** 10.1155/2012/541842

**Published:** 2012-07-19

**Authors:** Evgenia Halkia, John Spiliotis, Paul Sugarbaker

**Affiliations:** ^1^Department of Gynecology, Metaxa Cancer Memorial Hospital, 18537 Piraeus, Greece; ^2^1st Department of Surgical Oncology, Metaxa Cancer Memorial Hospital, 18537 Piraeus, Greece; ^3^Washington Cancer Institute, Program in Peritoneal Surface Malignancy, Washington, DC 20010, USA

## Abstract

The management and the outcome of peritoneal metastases or recurrence from epithelial ovarian cancer are presented. The biology and the diagnostic tools of EOC peritoneal metastasis with a comprehensive approach and the most recent literatures data are discussed. The definition and the role of surgery and chemotherapy are presented in order to focuse on the controversial points. Finally, the paper discusses the new data about the introduction of cytoreductive surgery and hyperthermic intraperitoneal chemotherapy (HIPEC) in the treatment of advanced epithelial ovarian cancer.

## 1. Introduction

Epithelial ovarian cancer (EOC) affects over 210,000 women and causes 128,000 deaths annually worldwide [1]. This cancer remains the leading cause of death from gynecology malignancy in the USA and was responsible for 14,600 deaths in 2009 [2]. The annual incidence and mortality rates have dropped 1.6% and 0.3% per year on average for the years 1997–2006 [3]. Current standard treatment of EOC is cytoreductive surgery (CRS) in order to remove the primary tumor and debulk any metastatic disease in combination with systemic chemotherapy with paclitaxel and platinum-based agents (carboplatin or cisplatin).

 Despite this treatment, only 46–49% of women with EOC will survive 5 years [4, 5]. While the incidence is low before the menopause, it rises after that with a median age at the time of diagnosis of 63 years. The lifetime risk of ovarian cancer is 1 in 70, but there are women with much higher risk especially those with germ line mutations of BRCA_1_ and BRCA_2_ tumor suppressor genes [6, 7].

 If there is a response to systemic chemotherapy, the disease often relapses within 12 to 18 months. The pattern of treatment failure is mostly local-regional, involving only the peritoneum and adjacent intra-abdominal organs. With this natural history, EOC patients may be candidates for local-regional in addition to systemic chemotherapy treatment [8].

## 2. Biology of Peritoneal Metastasis from**** Ovarian Cancer

 Malignancies that are managed as EOC may have as a primary site the epithelium of the ovary, the peritoneum itself (primary peritoneal adenocarcinoma), or the fallopian tube. They are histologically and clinically similar and are treated in the same fashion [9]. In this paper they are grouped together as EOC.

 EOC frequently spreads by direct extension from the primary site tumor to neighboring organs such as bladder and large bowel. Also, exfoliated tumor cells detach from the primary tumor and are transported throughout the peritoneal space by peritoneal fluid and disseminate within the abdominal cavity. Extensive seedy of the peritoneal cavity by tumor cells is often associated with ascites, particularly in advanced high-grade serous carcinomas. Usually patients with EOC have peritoneal deposits in the pelvis with contiguous extension to, or encasement of, the internal genitalia organs (uterus, fallopian tube, ovaries) and the rectosigmoid colon. Unlike other gynecologic cancers, EOC rarely disseminates through the bloodstream. However pelvic and/or para-aortic lymph nodes can be involved [10, 11]. The greater omentum has a large phagocytic capacity for cancer cells so that this organ is almost always infiltrated by the tumor [12].

### 2.1. Exfoliation of Epithelial Ovarian Cancer Peritoneal Metastases

 The biological behavior of the EOC is markedly different from the well-studied pattern of hematogenous metastasis found in most other cancers. The progression of metastases onto peritoneal surfaces appears to be very direct for ovarian cancer [12, 13]. After cancer cells have been detached from the primary cancer as single cells or clusters of cancer cells, they metastasize through a passive mechanism carried by the physiological movement of peritoneal fluid to peritoneal surfaces and omentum.

 An important molecule that helps the ovarian cells detach is *E-cadherin*, a membrane glycoprotein located within cell junctions [14]. In EOC peritoneal metastases, the E-cadherin expression of the ovarian cancer cells within peritoneal fluid is lower than in the primary tumor. This observation suggests that cells with low E-cadherin expression are more invasive and the absence of E-cadherin expression in ovarian peritoneal carcinomatosis predicts poor patient survival [15].

### 2.2. Epithelial Ovarian Cancer in Peritoneal Fluid

After the cancer cells detach, they float in the peritoneal fluid as single cells or as multicellular spheroids. Within the spheroids the cancer cells maintain a epithelial phenotype and express Sip 1, a regulator of E-cadherin and matrix metalloproteinase (MMP-2) [16]. In this phase, integrins (a_5_b_1_) and its ligands, fibronectin, are present on the surface of the cancer cells and play with other integrins (a_6_B_1_ and a_2_B_1_) an important role in spheroid growth and attachment. These molecules modify the microenvironment of ovarian peritoneal metastasis while in ascites fluid. This microenvironment provides the ovarian cells and spheroids the cell surface receptors to adhere to the peritoneal or omentum surfaces [17].

 Proteolytic activity is also very important during this journey of ovarian cells. Matrix metalloproteases as MMP-14 or MMP-2 possibly promotes the fast disaggregation of the spheroids to augment adhesion to the peritoneal surface mesothelial cell layer.

### 2.3. Epithelial Ovarian Cancer Implantation

The organ distribution of ovarian carcinoma metastasis is not random. Initial implantation is on the fallopian tube and the contralateral ovary. Then the most common sites for distant metastasis are the omentum and the peritoneum. The peritoneum beneath the right diaphragm and the small bowel mesentery are preferentially colonized [18].

 The mechanisms of cancer cell implantation are not yet well defined. Is it the primary ovarian tumor that prepares the omentum and peritoneum for successful colonization by secretion of factors? Are mobilized bone marrow cells recruited to prepare the metastatic site [12, 13]? Or is an interaction between the cancer cells and the mesothelial cells covering the basement membrane, which stimulates integrins, vascular adhesion molecules and CD44, the principal cell surface receptor for hyaluronic acid? As cancer cells adhere and invade, the mesothelium stimulates MMP2/9 to induce mesothelial cell apoptosis. This is promoted by secretion of Fas-ligand which then binds to a Fas receptor (CD 95) on mesothelial cells [19–21]. This process may be regulated by a protein, transglutaminase2, which is secreted in the ascites [22] and modulates the extracellular matrix of mesothelium.

### 2.4. Epithelial Ovarian Cancer Implant Progression

Little is known about progression of the ovarian cancer cells after implantation. The study of other cancers suggest that once the metastatic tumor reaches a certain size they require new blood vessels to provide nutrients for the growing tumor. In like manner for ovarian peritoneal metastases, the colony of ovarian cancer cells and spheroids attract new blood vessels to support their growth. A group of vascular endothelial growth factors (VEGFs) stimulate vascular and lymphatic endothelium to form new blood vessels to support their growth. These high levels of VEGFs in serum, ascites, and expression on ovarian carcinoma tissue have been associated with ovarian tumor progression and poor prognosis [23]. Recent studies with microarray demonstrate that the metastatic process in ovarian peritoneal metastasis require genetic changes present in the primary tumor [24].

## 3. Staging and Symptoms of Ovarian Peritoneal Metastases

### 3.1. Staging

**Figure 1:**
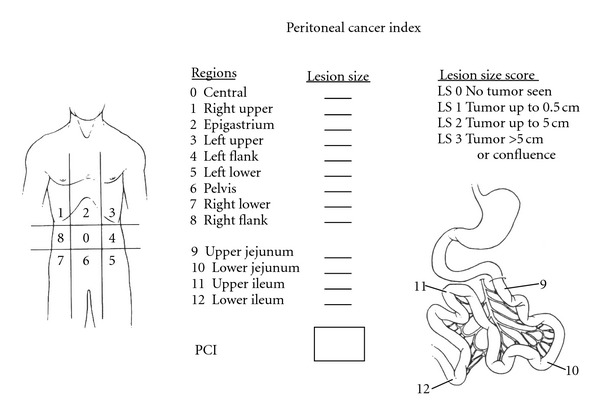
Peritoneal cancer index.

 Disease progression is described for all three types of ovarian cancer by both the TNM and FIGO staging systems [25, 26]. The stages associated with peritoneal metastases are FIGO III, which includes disease that has spread from the ovaries with visible peritoneal implants outside the pelvis (III_b_) and retroperitoneal lymph node involvement (III_c_). Stages III_b_ and III_c_ according to FIGO nomenclature represent 60% of cases of EOC [27]. For a description of the distribution and extent of metastases, one employs the peritoneal cancer Index (PCI) reported by Jacquet and Sugarbaker [28]. This index is a quantitative assessment of both cancer distribution and cancer implants size throughout the abdomen and the pelvis. Two components are involved in its calculation. One component is the distribution of the tumor in the abdominopelvic regions and the other is lesion size score (Figure 1).

## 4. Symptoms

The symptoms of peritoneal progression from EOC are often nonspecific and frequently caused by advance disease. Symptoms present are pelvic or abdominal pain, bloating, indigestion, abdominal distention, early satiety, and pain with intercourse. There is a symptom index in order to identifying women at risk to peritoneal carcinomatosis [29, 30]. It is not known if ascites is usually present when tumor cells initially metastasize or if ascites is a sign of a more advanced high volume disease. A combination of factors can contribute to ascites formation in ovarian cancer. Cancer cells can obstruct subperitoneal lymphatic channels and prevent the absorption of the physiologically produced peritoneal fluid. In addition, secretion of VEGF by ovarian cancer cells increases the vascular permeability and promotes the ascites formation [31, 32].

## 5. Diagnosis of Peritoneal Metastases from**** Ovarian Cancer

 The aim of the preoperative diagnostic assessment in patients with EOC is to estimate as accurately as possible the extent and anatomic location of disease.

### 5.1. Tumor Markers

While CA-125 (and other markers) are elevated in most patients with advanced disease, it is not specific for peritoneal carcinomatosis from EOC. CA-125 may be elevated in many other conditions. Also in the presence of ovarian cancer, CA-125 does not distinguish between localized or diffuse peritoneal disease [33–35]. Recent studies analyzed the serum cathepsin L (CL), heparane (Hpa) and MMg, and serum survivin for determining the degree of ovarian invasion and peritoneal metastases before surgery. The elevated levels of all of these are correlated with invasion and progression in ovarian cancer [36, 37]. Serial measurements of CA-125 are useful for monitoring for recurrent or metastatic disease provided that it was elevated prior to treatment and normalized during treatment.

### 5.2. Ultrasound

Ultrasound is a useful tool for the initial diagnosis in ovarian cancer. For determining the extent of peritoneal metastases, it is less accurate. It can detect ascites and splenic and liver metastasis, but it does not image peritoneal nodules accurately enough to evaluate the extent of the disease [35].

### 5.3. CT Scan

The role of CT in the preoperative evaluation of patients with ovarian cancer is controversial. Also the role of CT imaging in recurrent or peritoneal dissemination from ovarian cancer has received little attention and has not been clarified. The potential role of CT imaging to identify nonresectable disease in primary ovarian cancer has been shown [38]. However, the precise role for cross-sectional imaging has not been identified in the planning, monitoring of treatment response, or in assessment of chemotherapy-refractory or recurrent ovarian cancer. Recent studies attempt to correlate the CT findings with surgical outcome and PCI index to assist in identification of tumor respectability. CT scan seems to be helpful in patients with solitary site as the cause of bowel obstruction. On the other hand, successful treatment or palliation is still feasible in the presence of peritoneal metastases identified on CT scan. This finding alone should not be the reason to avoid surgery in well-selected patients [39]. Recently, the evaluation of multidetector CT (MDCT) in identifying peritoneal deposits preoperatively demonstrates that this procedure is useful in the assessment of the disease at specific locations in the abdomen and pelvis (pouch of Douglas and right subdiaphragmatic area) [40].

### 5.4. Magnetic Resonance Imaging

Magnetic resonance imaging (MRI) is becoming increasingly important in the diagnostic work up of EOC. MRI has demonstrated value in the evaluation of patients with advance disease. Some studies have shown that higher sensitivity may be achieved with oral contrast agents used for detection of peritoneal or omental dissemination [41]. Efforts in recent years have been focused on the design of systemic MRI contrast agents, which either target biomarkers or take advantage of the different physiology of cancerous cells.

 Diffusion-weighted imaging of peritoneal metastases of ovarian cancer is a functional MRI technique that exploits the restricted water mobility within hypercellular tumors to increase the contrast between these lesions and surrounding tissue [42]. Some groups suggest that this technology improves the detection and delineation of peritoneal implants at both initial staging and followup.

### 5.5. Positron Emission Tomography

Positron Emission Tomography (PET) imaging evaluates the biochemical and physiological characteristics of tumor cells, generating a radiographic picture of metabolic activity from the cancer nodule that is not possible with other imaging methods as CT or MRI.

 Increased accuracy of PET-CT on peritoneal metastases from ovarian cancer or the recurrence of ovarian cancer is apparent [43]. A recent report from Australia demonstrates that PET-CT scan [44]

- alters management in almost 60% of patients with peritoneal carcinomatosis from ovarian cancer,
- detects more sites of diseases than abdominal and pelvic CT,
- provides superior detection of nodal peritoneal and subcapsular liver disease,
- offers the opportunity for technology replacement in this setting.

When one compares contrast-enhanced CT, and PET-CT, there is a similar accuracy in detection of recurrent ovarian cancer [45].

## 6. Surgical Management of Peritoneal**** Metastases from Epithelial Ovarian**** Cancer

Cytoreductive surgery (CRS) may be considered for EOC is at the time of initial treatment (frontline) following neoadjuvant chemotherapy (interval debulking), and with recurrence [46, 47]. It has been established that improved survival following surgery is associated with minimal-volume residual disease. In Table 1, we list the possible indications and time points for surgical intervention in ovarian cancer [48].

 In the past, CRS with residual cancerous lesions >1 cm or <2 cm in greatest dimension was considered “optimal.” However, the precise definition of optimal or complete cytoreduction has been open to wide differences of opinion and has changed considerably over time. Optimal cytoreduction definitely improves the survival and requires peritonectomy procedures and visceral resections depending on the extent of peritoneal metastases [49–51]. After finishing the CRS, it is important to determine the completeness of cytoreduction score (CCs).

  CC-0 indicates no visible residual tumor.
  CC-1 indicates residual nodules <2.5 mm.
  CC-2 indicates residual nodules >2.5 mm and <2.5 cm.
  CC-3 indicates residual nodules >2.5 cm.

This score proposed by Sugarbaker and Chang has been accepted worldwide by the teams of peritoneal surface malignancy treatment groups [52]. 

### 6.1. Optimal Debulking

The phrase “optimal debulking” has been introduced for primary CRS. Retrospective studies reported a threshold of ≤1 cm of residual tumor as cut-off for inclusion criteria as complete cytoreduction [53, 54]. Nowadays, the definition of complete CRS has changed to indicate complete resection of all visible tumor, and the Gynecologic Cancer Interstudy Group (GCIG) has changed the official nomenclature to indicate this [55]. However, the concept of “optimal debulking” has not been established in CRS for recurrent disease.

 The incidence of patients with complete cytoreduction as defined above (CC score of 0 or 1) varied between 9 and 82% in a systematic review comprising retrospective studies with more than 20 patients [56] and between 9 and 100% in a meta-analysis published in 2009 [57]. Series including >100 patients with cytoreductive surgery for recurrent or peritoneal relapse showed controversial finding concerning the impact of the complete cytoreduction on survival. Some studies [56, 58, 59] reported a significant survival benefit only for patients with complete resection; others indicated a benefit also in patients with residual disease up to 0.5 cm or less than 1 cm [53, 60].

 A recent meta-analysis of several studies for surgery in recurrent disease or peritoneal metastases found that obtaining complete cytoreduction in an additional 10% of patients increased median survival by 3.0 months [57]. The first goal of surgery should be optimal CRS. However, if complete resection is not possible, the surgery may be modified in order to minimize surgical morbidity and mortality.

### 6.2. Predictors for Complete Cytoreduction in Ovarian Peritoneal Metastases

It is difficult to establish selection criteria for surgical intervention in ovarian peritoneal metastases. CA-125 elevation was found to be a predictive factor and the rate of complete resection declines by approximately 3% per week, after first CA-125 elevation was noticed and no surgery was performed [61]. Multivariate analysis of four retrospective studies demonstrated that absence of preoperative salvage chemotherapy, good performance status, and size of recurrent disease less than 10 cm were predictors for complete cytoreduction [58]. Also the number of diseases sites (solitary versus multiple) was an independent factor for complete cytoreduction [62]. Complete cytoreduction is not possible if distant or unresectable metastases are present or if small bowel is extensively seeded [63].

 The DESKTOP I trial conducted by the Arbeitsgemeinschaft Gynäkologische Onkologie (AGO) identified a combination of predictive parameters for complete resection: good performance status (ECOG), no residual disease after surgery for primary ovarian tumor or alternatively early initial FIGO stage, and absence of ascites by radiologic studies. Complete resection was achieved in 79% of patients scoring all these factors. If not all factors were positive, a complete resection was achieved in only 43% [64]. The latter group could be further differentiated: complete resection was achieved in 74% of this subgroup, if there were no peritoneal metastases found intraoperatively otherwise only 26% could be completely resected [65].

 In the DESKTOP II trial, the “AGO score” was validated in a prospective multicenter study. In 512 patients with primary disease, there were 261 patients (51%) with good performance status, complete resection at primary surgery, and absence of ascites and were defined as a positive “AGO score.” From these, 129 (49.4%) had a first relapse and underwent surgery for recurrent disease. These patients with a positive “AGO score” had a complete resection rate of 76% [66]. In conclusion, the “AGO score” may help to identify patients in whom complete resection of relapsed ovarian cancer is most likely.

### 6.3. Prognostic Factors Associated with Prolonged Survival in Patients Who Received Surgery in Recurrent or Advanced Ovarian Cancer

Many series reported a relationship between survival and surgical outcome. Complete cytoreduction was the strongest predictors for survival in all multivariate analyses performed. All other analyzed factors provided controversial results. Treatment-free interval between initial treatment and cytoreductive surgery showed no significant impact on outcome in univariate analysis in 50% of the series but others reported a significant role [56].

 The DESKTOP I trial showed a benefit for treatment-free interval exceeding 6 months but no differences if the interval was longer than 6 months. The same applies to the series of Chi et al., [67]. A similar observation was reported by Zang et al. who saw a benefit for longer progression-free intervals in univariate analysis, which could not be confirmed by multivariate analysis [60].

### 6.4. Lymph Node Metastases in Patients with Peritoneal Metastases from Ovarian Cancer

The presence of lymph node metastases in patients with advanced ovarian cancer or with peritoneal metastases indicates a poor prognosis. Its role in diagnosis is clear but its therapeutic role remains controversial, and the role for systematic removal of retroperitoneal lymph nodes as part of maximal cytoreduction is still unclear [68].

 A recent study from Italy [69] showed that the addition of systematic lymphadenectomy to cytoreductive surgery prolonged progression-free survival, which, in turn, may have an important impact on the quality of life of patients with advance disease. However, systematic lymphadenectomy did not prolong overall survival. The superior assessment of node status in patients undergoing lymphadenectomy could help refine the prognosis of patients with advanced ovarian cancer.

## 7. Morbidity and Mortality in Cytoreductive**** Surgery for Peritoneal Metastasis from**** Ovarian Cancer

 Postoperative morbidity and mortality rates are quite variable between institutions. Mean 30-day morbidity varies between 19.2% and 34% [57, 67, 70]. Complications rates in cytoreductive surgery for recurrent ovarian cancer are not significantly higher, compared to primary debulking surgery [71]. Mean 30-day mortality rate ranges between 0.7 and 2.8% for primary debulking surgery, while the mortality rate of surgery in recurrent disease range between 1.2 and 5.5% [57, 59, 66, 72].

### 7.1. Long-Term Systemic Plus Intraperitoneal Chemotherapy for Treatment of Primary Disease

Intraperitoneal chemotherapy (IP) is designed to improve the pharmacokinetic profile of chemotherapeutic agents and thereby deliver higher doses into the anatomic compartments that are at greatest risk for disease recurrence. The majority of IP chemotherapy solution stays within the peritoneal compartment, with limited deep tissue penetration; therefore, it is indicated only for patients who have completed cytoreductive surgery in combination with IV chemotherapy as initial treatment with a significant benefit in overall survival 65.5 months in the IV + IP arm versus 49.7 months in the IV only arm [73]. Studies in recurrent ovarian cancer after secondary cytoreductive surgery are needed in order to identify the possible benefit of this strategy for recurrent disease. The German Association of Gynecologic Oncology (AGO) has now initiated a study in advanced ovarian cancer (LION), which compares the value of systematic lymph node dissection with no lymph node resection in patients without any visible tumor residuals (NCT00712218). Until these data are in fact available, patients with advanced ovarian cancer should be informed in detail about the pros and cons of systematic lymph node dissection.

## 8. Systemic Chemotherapy for Recurrent**** Disease

While increasing numbers of patients with ovarian cancer are experienced 5-year survival, 90% of suboptimally debulked patients and 70% of optimally debulked patients relapse 18 to 24 months following primary treatment [73, 74]. Traditionally patients with recurrent platinum-sensitive ovarian cancer, defined as a disease-free interval from completion of primary treatment of at least 6 months, have been retreated with platinum-based chemotherapy, often in combination with another cytostatic agent.

 In ICON 4 study patients with recurrent disease were randomized to receive a platinum-based regimen with or without a taxane. In the taxane-containing arm, 90% received paclitaxel as a part of a doublet. Results demonstrated that patients in the taxane group experienced higher response rate, longer progression-free survival, and superior overall survival compared to those who received retreatment with single-agent platinum [75]. A major problem in retreatment is the cumulative toxicity from primary therapy.

 Another study, AGO OVAR 2.5, compared single-agent carboplatin with the combination regimen of gemcitabine and carboplatin in recurrent disease. The study showed that double drug treatment experienced a higher response rate and a superior progression-free survival but not difference in overall survival and concluded that the doublet of gemcitabine and carboplatin was an acceptable regimen for recurrent disease [76]. Currently, the OCEANS trial is evaluating outcomes of previous doublet drugs in combination with bevacizumab [77].

 As an alternative strategy, the CALYPSO trial randomized patients to receive either the doublet of pegylated liposomal doxorubicin (PLD) and carboplatin versus paclitaxel and carboplatin [78]. The study demonstrated an improvement in progression-free survival for the PLD/carboplatin arm (median 11.3 months versus 9.4 months, *P* < 0.005) with less marrow toxicity and carboplatin hypersensitivity reactions.

 Whereas combination treatment with platinum doublet is frequently used for recurrent platinum-sensitive patients, single-agent treatment is currently the preferred approach for platinum-resistant patients or for platinum-sensitive patients who have a short time to recurrence, such as a 6- to 12-month disease-free interval [79]. Numerous agents are available that can be used as single-agent therapy—gemcitabine, PLD, topotecan, paclitaxel, docetaxel, oral etoposide, and hormonal agents. Also worthy of consideration is the patients anticipated tolerability and cumulative toxicity from the frontline therapy in making the individual treatment selection for recurrent disease.

## 9. Target Therapies for Recurrent Disease

Targeted therapeutic agents are currently analyzed in clinical trials to evaluate translational end points in order to select patients and monitoring therapeutic response.

### 9.1. Antiangiogenic Agents

 Numerous protocols evaluating antiangiogenic agents in combination with cytotoxic chemotherapy for recurrent disease are currently open [80]. The use of bevacizumab in recurrent ovarian cancer has been explored with promising results and response rates up to 24% [81].

### 9.2. mTOR Inhibitors

Many mTOR inhibitors are in clinical trials. GOG 1701, a phase II study for recurrent/persistent ovarian cancers, evaluated the use of temsirolimus in recurrent ovarian cancer and primary peritoneal cancer. Results presented in 2010 suggested modest activity of weekly single-agent temsirolimus in persistent or recurrent disease, with 24.1% progression-free survival ≥6 months [82].

### 9.3. PARP Inhibitors

Inhibition of polyAdenosine diphosphate-ribose polymerase (PARP), a key enzyme in the repair of DNA, may lead to the accumulation of breaks in double-stranded DNA and cell death. A phase II study with these inhibitors demonstrated a clinical benefit in the 57.6% of patients with platinum-sensitive ovarian cancer as a treatment in recurrent disease [83].

### 9.4. Histone Deacetylase Inhibitors

A phase II study by the GOG (protocol 0126T) is examining the use of belinostat in combination with carboplatin among patients with recurrent or persistent platinum-resistant disease. Histone hypoacetylation has been associated with malignancy through the transcriptional silencing of tumor suppressor genes [84].

## 10. Hyperthermic Intraperitoneal**** Chemotherapy in Peritoneal Metastases ****from Epithelial Ovarian Cancer

 The first report of the use of hyperthermic intraperitoneal chemotherapy (HIPEC) for EOC was in 1994 [85]. Since that time, there has been a large volume of published research evaluating this modality in conjunction with CRS. The published reports are mainly case series and early phase II studies. The patients are in variable stages of their disease with HIPEC used as frontline treatment, interval debulking treatment, or as adjuvant treatment in recurrent disease. Recently Spiliotis et al. [86] in a small phase III prospective trial evaluated the role of CRS and HIPEC plus systemic chemotherapy versus CRS plus systemic chemotherapy in women with recurrent EOC after initial debulking surgery and systemic chemotherapy. The median survival rate was 19.5 months versus 11.2 months (*P* < 0.05) and the three-year survival was 50% versus 18% in favor of the HIPEC group [86].

 HYPER-O, an internet registry, collected and analyzed data from multiple centers to achieve an understanding of current practice and outcome [87]. In the initial report, 141 women were treated; as frontline (*n* = 26), as interval debulking (*n* = 19), for consolidation (*n* = 12), or for recurrence (*n* = 83). The median duration of HIPEC was 100 min (range 30–120), the average perfusion temperature was 38.5–43.6°C (median 41.9°C). The HIPEC drug was with platinum (*n* = 72), mitomycin (*n* = 53), or a combination (*n* = 14). The median overall survival was 30.3 m.

 The results of HYPER-O study are presented in Table 2.

### 10.1. HIPEC as Frontline Treatment

The evolution of management of advanced EOC in the last decade has been characterized by the validation of intraperitoneal chemotherapy. A Cochrane meta-analysis of all randomized intraperitoneal versus intravenous trials showed a hazard ratio, 0.79 for disease-free survival and 0.79 for overall survival favoring in the intraperitoneal arms [88]. The use of HIPEC as frontline treatment is presented in several studies with small number of patients. The data suggests that with HIPEC 2-year overall survival and progression-free survival were not significantly different with those of cytoreductive surgery and systemic chemotherapy. Rufian et al. reported 19 patients with stage III cancer treated at the time of frontline surgery with paclitaxel for 60 minutes at 41–43°C [89]. The mean overall 3- and 5-year survival was 46 and 37%. In patients with complete cytoreduction, there was a median overall survival of 66 months. Similar results were demonstrated recently by Deraco and coworkers [90]. These results are comparable but do not exceed studies with maximal CRS followed by systemic chemotherapy in frontline treatment of EOC.

### 10.2. Use of HIPEC during Interval Cytoreduction

A major controversy concerns the optimal time-point in the natural history of EOC for the performance CRS + HIPEC [91]. Data suggests that maximal surgical effort, combined with systemic and intraperitoneal chemotherapy in the primary setting, represents indirect evidence that CRS + HIPEC could be tested as upfront treatment in the context of a phase III trial [92]. The use of CRS following the maximal response from neoadjuvant systemic chemotherapy is theoretically the most optimal time-point for HIPEC [92].

 The numbers from different studies and especially from HYPERO are small and the data difficult to interpret. When one compares the survivals between patients when HIPEC used as frontline or used at the time of interval debulking following neo-adjuvant chemotherapy, there was no significant difference [87]. However, a large randomized study showed no difference in overall survival in women with stage IIIc and IV disease randomized to initial CRS then intravenous chemotherapy or neo-adjuvant chemotherapy followed by interval debulking surgery then further systemic chemotherapy [93]. Recently, Spiliotis et al. reported an ongoing trial of laparoscopic-assisted neoadjuvant HIPEC in patients with stage IIIc or IV ovarian cancer, in combination of systemic chemotherapy followed by interval debulking + HIPEC and then further systemic chemotherapy [94].

### 10.3. HIPEC in Recurrent EOC

Survival for patients with recurrent EOC, treated by chemotherapy alone, tends to be inferior to that reported for secondary CRS. The influence of secondary CRS without HIPEC on survival outcomes has been addressed in a substantial number of studies and has been recently systematically reviewed [95]. However, these were noncontrolled studies not strictly comparable since chemotherapy trials will include patients not suitable for traditional cytoreduction including patients with a high PCI. A consistent survival data comparing secondary CRS with chemotherapy is expected to be provided by the ongoing randomized trial AGO-OVAR OP4 [96].

 Results from studies reporting median and mean overall survival and progression-free survival are given in Table 3 [86, 97–103]. These data suggest that HIPEC is an interesting and promising treatment in recurrent EOC when it is combined with complete cytoreduction. The numbers are small but interesting in that the 3-year and 5-year survivals were significantly better in the HIPEC group versus conventional treatment [101–103].

 The prognostic factors, which can predict the survival outcome, define also the criteria for “optimal”-HIPEC in recurrent ovarian cancer [86, 104]. These are age, performance status, interval from initial treatment to recurrent, PCI, completeness of cytoreduction, presence of lymph nodes, and initial platinum response (Table 4).

### 10.4. HIPEC as Consolidation Treatment

Consolidation treatment is defined as additional treatment following a complete response to frontline therapy. Patients with initial stage III EOC were treated with HIPEC at second laparotomy compared with patients who had a complete response but did not receive HIPEC [105]. The 5-year survival rate was 66.1% with HIPEC versus 31.3% in the control group.

 In another study of 51 patients with EOC underwent frontline surgery with CRS and systemic chemotherapy and a CC-0/CC-1 cytoreduction. Thirty-two underwent second-look laparotomy with HIPEC and the others 19 who refused second look were used as a control group. The median survival was 64.4 months in HIPEC arm versus 46.4 months in control group [106]. A future project is to use HIPEC consolidation treatment in second-look laparoscopy in order to reduce the surgical morbidity.

### 10.5. Morbidity and Mortality of HIPEC

There is a question that arises when discussing the morbidity and mortality in this treatment. It is unclear whether increased morbidity and mortality is related to CRS or to HIPEC. The estimation of morbidity and mortality related to HIPEC delivery is complicated by the fact that the major surgery with visceral resections and peritonectomy procedures is itself associated with high morbidity. In a recent study by Fagotti et al., in recurrent ovarian cancer with CRS and HIPEC, the morbidity rate was 34.8% with no mortality. Ileus, anastomotic leakage, bleeding, wound infection, fistula formation, pleural effusion, and thrombocytopenia represented the commonest complications [107].

 Postoperative bleeding is a serious complication especially if oxaliplatin is used for HIPEC. One study reported premature closure because of a 29% severe morbidity rate [108]. The rate of anastomotic leak in the absence of a diverting stoma remains unknown and range between 1.6% and 3% [109]. Spontaneous bowel perforation may reflect the effect of heated chemotherapy on bowel, which has been traumatized during the enterolysis.

 Hematological complications due to HIPEC are common and are a drug-dependent complication. The morbidity and mortality in patients with EOC having CRS and HIPEC remains dependent upon the patient's age and performance status, the number and type of peritonectomy procedures, and the duration of HIPEC.

An important factor to reduce the morbidity and mortality in cytoreductive surgery and HIPEC is the importance of learning curve. The performance of at least 130 procedures is necessary to consider the physician an expert in cytoreduction using the Sugarbaker technique [110].

## 11. Conclusions

Peritoneal metastases in patients with EOC are a poor prognostic factor for survival. An optimal management strategy includes CC-0/CC-1 CRS, but the role of HIPEC in this disease remains level 4 [111]. Innovative clinical studies with sufficient data need to compare conventional treatment with and without HIPEC [111].

A problem in the evaluation of HIPEC for the treatment of ovarian cancer concerns the adequacy of the HIPEC chemotherapy regimen. In many instances mitomycin C alone has been used. In other HIPEC chemotherapy regimens, it has been moderate dose cisplatin combined with doxorubicin. To this point in time, no large phase II trials using bidirectional chemotherapy at maximum doses has been used. Also, HIPEC has not been combined with EPIC in order to maximize the perioperative use of paclitaxel. Paclitaxel is usually used as EPIC at moderate dose for 5 days postoperatively. Phase II trials with a more modern perioperative chemotherapy regimen that would have a higher response rate need to be performed. The perioperative chemotherapy must be effective enough to maintain the surgical complete response that can be achieved with an optimal cytoreduction using both peritonectomy and visceral resections.

In the future, understanding both genome structure variation and functional deregulation in cancer may predict which patients with EOC are candidates to develop peritoneal metastases and which patients will be benefitted by selected chemotherapy agents [112].

## Figures and Tables

**Table 1:** Indications for surgery in ovarian cancer.

(i) Diagnostic laparotomy or laparoscopy	Exploration performed at any time in the course of ovarian cancer to obtain a histological diagnosis.
	*A second-look surgery *is performed in patients who are clinically, biochemically, and radiologically free of disease after completion of chemotherapy with the purpose to confirm the response status.
(ii) Staging laparotomy	Surgery performed in patients with clinically early ovarian cancer aiming at the detection of tumor spread.
(iii) Primary cytoreductive surgery	Surgery with the aim of complete resection of all macroscopic tumor in patients with first diagnosis of advanced ovarian cancer before any other treatment (e.g., chemotherapy).
(iv) Secondary surgery/Interval debulking	Surgery performed in patients usually after 3 cycles of chemotherapy, with an attempt to remove any remaining tumor, which has not been eradicated by chemotherapy.
(v) Surgery for progressive ovarian cancer	Surgery with the purpose of removing obviously resistant tumors, which have not responded to chemotherapy and progressed during primary chemotherapy.
(vi) Surgery for recurrent ovarian cancer	Surgery aiming for complete resection for all macroscopic tumor in patients with recurrent ovarian cancer after completion of primary therapy including a subsequent period without any signs of disease.
(vii) Palliative surgery	Surgery performed in patients with symptoms caused by progressive disease or sequelae aiming to relieve symptoms and not towards survival prolongation.

**Table 2:** Survival rates in HYPERO study. Adapted from [87].

Time-point HIPEC used	*n*	OS (m)	2 years %	5 years %
Overall	141	30.3	49.1	25.4
Frontline	26	41.7	57.0	33.3
Interval debulking	19	68.6	80.4	50.2
Consolidation	12	53.7	63.6	42.4
Recurrence	83	23.5	40.9	18.0

OS: overall survival.

**Table 3:** Cytoreductive surgery and hyperthermic intraperitoneal chemotherapy in recurrent epithelial ovarian cancer.

Author	Year	*N*	OS	(months)	PFS	(months)
			Median	Mean	Median	Mean
Deraco et al. [97]	2001	27			21.8	
Zanon et al. [98]	2004	30	28.1			
Raspagliesi et al. [99]	2006	40		41.4		23.9
Helm et al. [100]	2007	18	31		10	
Di Giorgio et al. [101]	2008	25	22.5		15.5	
Fagotti et al. [102]	2009	25			10	
Carrabin et al. [103]	2010	8			10	
Spiliotis et al. [86]	2011	25	19.5		14.5	

OS: overall survival; PFS: progression-free survival.

**Table 4:** Prognostic-predictive factor for “optimal” HIPEC in recurrent EOC.

(i) Age < 65
(ii) Performance status >80
(iii) Interval from initial diagnosis >12 months
(iv) Peritoneal Cancer Index <20
(v) Completeness of Cytoreduction CC-0 or CC-1
(vi) Absence of retroperitoneal lymph nodes
(vii) Platinum-sensitive
